# Lipid Metabolism Regulation Based on Nanotechnology for Enhancement of Tumor Immunity

**DOI:** 10.3389/fphar.2022.840440

**Published:** 2022-03-22

**Authors:** Bin Tu, Yanrong Gao, Feifei Sun, Mingjie Shi, Yongzhuo Huang

**Affiliations:** ^1^ State Key Laboratory of Drug Research, Shanghai Institute of Materia Medica, Chinese Academy of Sciences, Shanghai, China; ^2^ University of Chinese Academy of Sciences, Beijing, China; ^3^ School of Life Science, Hangzhou Institute for Advanced Study, University of Chinese Academy of Sciences, Hangzhou, China; ^4^ Zhongshan Institute for Drug Discovery, SIMM, CAS, Zhongshan, China; ^5^ NMPA Key Laboratory for Quality Research and Evaluation of Pharmaceutical Excipients, Shanghai, China; ^6^ School of Advanced Study, Institute of Natural Medicine and Health Product, Taizhou University, Taizhou, China

**Keywords:** tumor metabolism, lipid metabolism, tumor immunity, tumor microenvironment, nanotechnology, cancer nanomedicine

## Abstract

The hallmarks of cancer include dysregulated metabolism and immune evasion. As a basic way of metabolism, lipid metabolism is reprogrammed for the rapid energy and nutrient supply in the occurrence and development of tumors. Lipid metabolism alterations that occur in the tumor microenvironment (TME) affect the antitumor responses of immune cells and cause immune evasion. Therefore, targeting lipid metabolism in the TME for enhancing the antitumor effect of immune cells is a promising direction for cancer treatment. Cancer nanomedicine has great potential in regulating tumor metabolism and tumor immunity. This review summarizes the nanotechnology-based strategies for lipid metabolism regulation in the TME for enhanced anticancer immune responses.

## Introduction

Mutations that drive tumorigenesis cause metabolic alterations (Min and Lee, 2018). The metabolism alteration has been considered an essential hallmark of tumorigenesis (Hanahan and Weinberg, 2011; Pavlova and Thompson, 2016). Lipid metabolism, one of the main sources of energy and basic component for living cells, is reprogrammed to meet the need for energy and nutrient supply to support the rapid growth of tumor cells. In tumor cells, the lipid uptake and synthesis are different from those in normal cells because of the reprogrammed metabolism. For example, tumor cells with RAS mutations can enhance lipid synthesis by upregulating the activity of phosphoenolpyruvate carboxykinase 1 (PCK1) (Xu et al., 2020). Cholesterol is an essential lipid of the cell membrane and a basic component for cancer cell proliferation. Cholesterol is also involved in intratumoral steroidogenesis and vitamin D synthesis, and serves as a signal molecule (e.g., estrogen-related receptor Alpha (ERRα) ligand) (Chimento et al., 2018). Cholesterol metabolism reprogramming in cancer changes the availability and usability of intratumoral cholesterol, and the cholesterol-derived metabolites contribute to cancer progression and immunosuppression (Huang et al., 2020). Lipid metabolism alteration occurs in both cancer cells and nonmalignant cells (e.g., stromal cells and immune cells) in the tumor microenvironment (TME). In the TME, the matrix compounds can provide raw materials for tumor cells to quickly synthesize the necessary molecules. It has been reported that under the conditions of local hypoxia and lipid depletion, acetate can serve as an alternative source for lipid synthesis in cancer cells (Comerford et al., 2014). Moreover, tumor-associated stromal cells can also provide nutrients for tumor development. For instance, tumor-associated fibroblasts could transport lipids to adjacent cancer cells through cargo vesicles for maintaining their fast proliferation (Santi et al., 2015).

Immunotherapy, representing a breakthrough in the cancer field, has been widely applied in the treatment of various cancers, including metastasis and advanced cancers, by activating or normalizing the immune system. However, immune evasion typically leads to poor anti-tumor effects of immunotherapy (Beatty and Gladney, 2015). Lipid metabolism alteration not only plays an important role in cancer cell proliferation and migration but also affects the recruitment and function of immune cells. Lipid metabolism abnormality in the TME is an important mechanism leading to immune evasion. For example, lipid accumulation in myeloid-derived suppressor cells (MDSCs), dendritic cells (DCs), tumor-associated macrophages (TAMs), and CD8^+^ T cells have been demonstrated to skew these immune cells towards immunosuppressive and immune-escaping phenotypes in the TME (Herber et al., 2010; Al-Khami et al., 2017; Ma et al., 2019; Rabold et al., 2020; Su et al., 2020). Therefore, targeting lipid metabolism is a potential strategy for tumor immunotherapy, which has been reviewed by Bian, et al. (2021); Broadfield, et al. (2021).

However, it remains a challenge to achieve precise regulation of intratumor lipid metabolism because of the complexity and heterogeneity of the TME. Furthermore, the lipid metabolism alteration in various tumors is different because of various genetic backgrounds and tissue origins, which has been described in a review (Bi et al., 2018). The fast-cycling tumor cells rely on aerobic glycolysis for energy supply, while the slow-proliferation cancer cells under glucose-deprived conditions use lipid oxidation as the main metabolism pattern (Hoang-Minh et al., 2018). Nanotechnology, which is emerging as a promising therapeutic tool for targeting some specific tissues or cells, has been actively explored for application in anticancer immunity enhancement *via* regulating metabolism, e.g., glycolysis (Wang et al., 2019; Chen et al., 2020) and lipids (Jin et al., 2019; Kim D. et al., 2021). Therefore, nanotechnology-based targeting delivery may provide a potential method for cell-targeting regulation of lipid metabolism in the TME for improving immunotherapy. Herein, we will summarize nanotechnology-based therapeutic strategies for reprogramming lipid metabolism and enhancing tumor immunotherapy.

## Lipid Metabolism Alternations in TME Suppress Tumor Immunity

The TME is characterized by hypoxia and glucose deprivation. To overcome the nutrient-deficient environment, the tumor cells develop survival mechanisms *via* metabolic reprogramming. Different from glycolysis, the diversity of structure and characteristics of lipid species may lead to the complexity of lipid metabolism in cells. It was found that the commonly changed lipid metabolism and corresponding pathways in tumors included fatty acids metabolism, cholesterol metabolism, and arachidonic acid metabolism, and PPAR (peroxisome proliferator-activated receptor) signaling by the analysis of various omics data (Hao et al., 2019). Meanwhile, these changes also play an important role in coordinating the immune cells toward an immunosuppressive phenotype.

## PPAR Signaling

**FIGURE 1:**
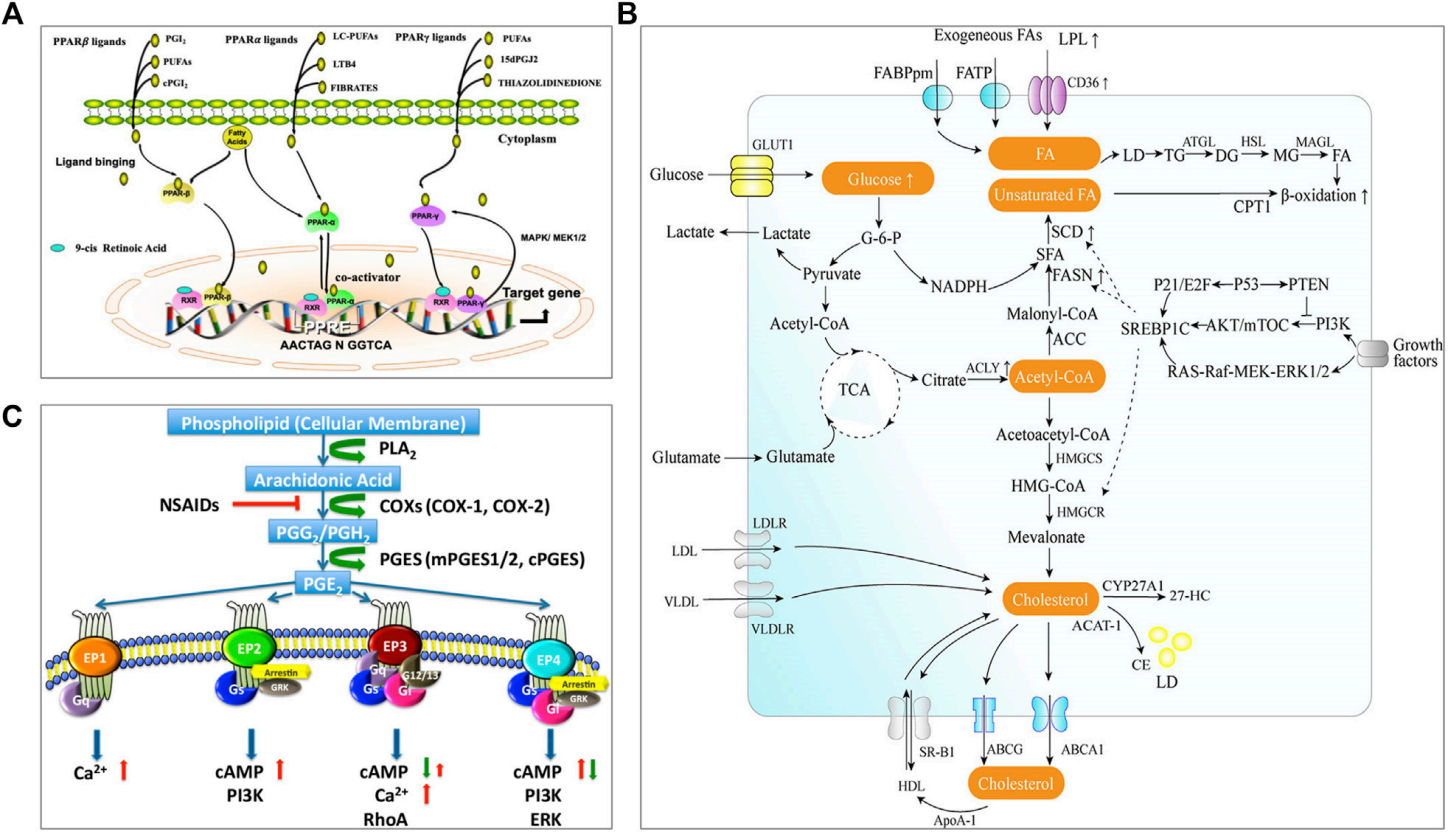
Lipid metabolism and pathways closely related to the occurrence and development of tumors. **(A)** PPAR signaling (Peng et al., 2021). The complete transcriptional activity of PPAR requires the binding of homologous lipid ligands. Endogenous PPAR ligands are originated from the nuclear membrane or transferred from the cytoplasm. PPAR can shuttle between the nucleus and cytoplasm, and it is mainly located in the nucleus. Ligand binding promotes PPAR conformational changes, enabling PPAR to interact with another nuclear receptor (retinoic acid IDX receptor, RXR) to form heterodimerization. The combination of PPAR heterodimer with PPAR response element (PPRE) in the target gene promoter activates the expression of related genes. **(B)** The metabolism pathway of fatty acid and cholesterol (Wang et al., 2020). There are two sources of intracellular fatty acids: intake of exogenous fatty acids and *de novo* synthesis of fatty acids. Exogenous fatty acids can be absorbed by cells through specific transporters, e.g., CD36, FATP, and FABPpm. Endogenous fatty acid synthesis begins with the product of acetyl-CoA from the tricarboxylic acid cycle (TCA) and depends on the activity of fatty acid synthase (FAS). The intracellular fatty acids are stored in lipid droplets (LD). Fatty acids will be catalyzed through mitochondria β-oxidation to produce energy. Intracellular cholesterol homeostasis mainly includes the regulation of *de novo* synthesis, uptake, storage, and efflux. Cells can absorb exogenous cholesterol through receptors, e.g., LDLR and VLDL. Endogenous cholesterol is synthesized by the mevalonate pathway. Excessive cholesterol in the cells will be transported outside through transporters, e.g., ABCA1, ABCG1, or converted into cholesterol esters by ACAT1 catalysis and stored in a form of lipid droplets. Cholesterol can be converted into cholesterol derivatives under the action of related enzymes e.g., CYP27A1 to support life activities. **(C)** Arachidonic acid and PGE2 signaling (Li et al., 2017). Arachidonic acid (AA) is a polyunsaturated fatty acid that forms the phospholipid domain of cell membranes and could be released from the cell membrane by cytoplasmic phospholipase A2 (PLA2). Free AA is metabolized to PGG2 and PGH2 by cyclooxygenase-1 (COX-1) or COX-2. PGH2 is relatively unstable and successively metabolized to PGE2 by cPGES and mPGES1/2. PGE2 could bind to four GPCRs (EP1-EP4). Different receptors are coupled to different signaling pathways. Reprinted under the Creative Commons CC BY license.

PPARs serve as lipid sensors in response to the increased energy requirement and are fatty acid-activated transcription factors, belonging to the nuclear hormone receptor superfamily with three nuclear receptor isoforms, PPARα, PPARβ/δ, and PPARγ **(** Figure 1A) (Peng et al., 2021). Lipid-derived metabolites serve as the natural ligands of PPARs. The tissue distribution of PPAR subtypes and their endogenous ligands and target genes are summarized in a review by Monroy-Ramirez, et al. (Monroy-Ramirez et al., 2021). PPARα is a regulator of fatty acid metabolism and involved in substrate transport, substrate oxidation, and oxidative phosphorylation (OXPHOS); PPARδ plays a vital role in fatty acid oxidation and blood glucose regulation; PPARγ can facilitate energy storage by promoting adipogenesis and lipid synthesis in different cell and play a major role in adipocyte differentiation (Christofides et al., 2021). The reprogrammed lipid metabolism and the resultant metabolites could affect tumor immunity *via* the PPAR signaling. For example, PPARδ signaling increased the contents of neutral lipid and membrane cholesterol in malignant B cells, and caused STAT3 activation and weakened immunostimulatory interferons (IFNs) signaling responses; therefore, PPARδ inhibition could promote the antitumor immune activity of IFNs (Sun et al., 2018).

## Alterations of Fatty Acid Metabolism in the TME

Both fatty acids and cholesterol are *de novo* synthesized from acetyl CoA from the tricarboxylic acid cycle (TCA) (as illustrated in Figure 1B, Wang et al., 2020). Fatty acid metabolism mainly includes *de novo* synthesis, oxidation, desaturation, and extension of fatty acids. Tumor cells are characterized by abnormal energy metabolism and up-regulated endogenous fatty acid synthesis (Rohrig and Schulze, 2016). Exogenous intake and *de novo* synthesis of fatty acids could be increased to meet the insistent need of tumor cells to produce plasma membrane phospholipids and lipid-based signaling molecules. Meanwhile, fatty acid metabolism is also changed in the intratumoral immune cells. For instance, an increased rate of fatty acid synthesis was found in the tumor-infiltrating Tregs, which could contribute to the Treg expansion in the TME (Pacella et al., 2018). Natural killer (NK) cells with the upregulated PPARα-mediated fatty acid catabolism showed impaired antitumor function (Michelet et al., 2018). In addition, it was found that the fatty acid oxidation (FAO) in the RIPK3-deficient TAMs could increase because the RIPK3-mediated ROS–caspase 1 suppression resulted in the activated PPARα/γ pathway, thus leading to TAM accumulation and M2 differentiation in TME during hepatocarcinogenesis (Wu et al., 2020). However, in breast cancer, the truncated PPARγ caused by capspace-1 mediated cleavage could attenuate MCAD activity and inhibit FAO; as a result, the consequent lipid droplet accumulation in TAM promoted the differentiation of TAMs to a pro-tumor phenotype (Niu et al., 2017). It indicated either activated PPARγ or truncated PPARγ could promote the pro-tumor effect of TAMs, but the further investigation should be carried out.

## Alterations of Cholesterol Metabolism in the TME

Cholesterol homeostasis is important to maintain the normal functions of the cell membrane and is regulated by *de novo* synthesis, import, and efflux [as illustrated in Figure 1B (Wang et al., 2020)]. Cholesterol level is typically enhanced in tumor tissues/cells, in which cholesterol synthesis may be up-regulated through PI3K/AKT/mTOR signaling or TP53 [see the review of (Kuzu et al., 2016)]. A study revealed that there was about 3-fold higher cholesterol level in tumor tissue compared to the normal (10.7 vs. 3.6 μmol/g) (Yoshioka et al., 2000). Cholesterol is involved in tumor-associated-macrophage polarization. The increase of cholesterol efflux from macrophages in the TME can activate the IL-4 signaling pathway and downregulate the IFN-γ transcription, thus inducing macrophages to a tumor-promoting immunosuppressive phenotype (Goossens et al., 2019). After homing to the tumors with rich cholesterol, T cells are prone to be exhausted due to increased cholesterol uptake and accumulation (Ma et al., 2019). Meanwhile, the impaired synthesis of cholesterol can diminish the anticancer ability of intratumoral invariant natural killer T (iNKT) cells; for example, the overproduced lactic acid in the TME can cause insufficient cholesterol synthesis in the iNKT cells by suppressing PPARγ expression, and thereby reduced IFN-γ production (Fu et al., 2020). Besides, cholesterol derivatives, e.g., oxysterols, also play a certain role in regulating the tumor immune microenvironment. Oxysterol is the oxidized form of cholesterol and shows significant enrichment in the TME (Kloudova et al., 2017). The enriched oxysterol in the TME can not only increase the recruitment of neutrophil (Raccosta et al., 2013) and MDSC (Baek et al., 2017) but also downregulate the antigen-presenting function of DCs (Villablanca et al., 2010) and the effector function of CD8^+^ T cells (Baek et al., 2017), resulting in the tumor immune evasion. In addition, macrophages may also be regulated by oxysterols to promote tumor cell metastasis (Eibinger et al., 2013).

## Alterations of PGE2 Produced by Arachidonic Acid Metabolism in the TME

Prostaglandin E2 (PGE2) is produced by arachidonic acid metabolism and is synthesized by cyclooxygenase (COX) and prostaglandin synthase [as illustrated in Figure 1C (Li et al., 2017)]. PGE2 is transported to the extracellular space through specific prostaglandin transporters or multidrug resistance-associated proteins (MRP) (Elwakeel et al., 2019). Extracellular prostaglandins activate four specific G protein-coupled receptors (GPCR), EP 1–4, for regulating the physiological activity of cells. COX is overexpressed in many tumors and is associated with immunosuppression accompanied by the high production of PGE2 in the TME (Zelenay et al., 2015). PGE2 overexpression in the TME can promote TAM to differentiate into a pro-tumor subtype and enhance the immunosuppressive functions of MDSCs (Obermajer et al., 2012; Larsson et al., 2015). Furthermore, the excessive production of PGE2 in the TME promotes the development and differentiation of Treg cells (Sharma et al., 2005). At the same time, the abundant PGE2 in the TME impairs the function of DC and NK cells, and abrogates the activation of CD8^+^ T cells, contributing to the tumor immune evasion (Ahmadi et al., 2008; Bottcher et al., 2018).

## Nanotechnology-Based Lipid Metabolism Regulation for the Enhancement of Tumor Immunity

Alterations of lipid metabolism in the TME play a significant role in the formation of tumor immunosuppressive microenvironment and lead to tumor immune evasion. To enhance tumor immunity *via* regulating lipid metabolism in the TME is a very promising direction for cancer treatment. However, it is difficult for precise regulation of lipid metabolism in the TME because of the obstacles against efficient drug delivery in the complex and heterogenetic TME to reach the target cells. Nanotechnology has recently drawn great attention for its application for remodeling TME (Fernandes et al., 2018; Sun et al., 2020). Nanotechnology-based drug delivery can significantly prolong the drug circulation time and improve bioavailability. It is an attractive strategy to effectively regulate lipid metabolism in the TME to enhance tumor immunity.

Notably, the nanomaterials could affect the lipid metabolism and functions of cells (Kim S. J. et al., 2021; Florance et al., 2021). Therefore, the biological effects of nanoparticles should be further studied, and this part will not be discussed in the review.

### T Cell-Targeted Nanotechnologies for the Enhancement of Tumor Immunity

Infiltrating T lymphocytes in the TME play a critical role in the cancer-immune surveillance and antitumor immune responses, and their differentiation and functions may be regulated by the TME metabolites derived from glucose and lipid metabolism (Liu et al., 2022). In the TME, efficient T cell responses against tumors are hampered by many factors, including immunosuppressive cells, immunosuppressive cytokines, tumor-derived inhibitory signals (e.g., PD-L1), the low immunogenicity of tumor cells, hypoxia, and nutritional deficiency (Anderson et al., 2017). CD8^+^ T cells are the main cytotoxic effector subset of tumor-infiltrating T lymphocytes. The deficiency of CD8^+^ T cell responses against tumors is a main cause for tumor immune evasion (Kim et al., 2007). The metabolic switch in the TME leads to the dysfunction of CD8^+^ T cells and is contributes to immune evasion (Fischer et al., 2007; Scott and Cleveland, 2016; Daneshmandi et al., 2019). Hypoxia in the TME induces the transformation of tumor cell metabolism from oxidative phosphorylation to glycolysis, which causes more consumption of carbohydrates rather than lipids. On the contrary, when subjected to hypoxia and hypoglycemia at the same time, the CD8^+^ T cells mainly rely on fatty acid oxidation in the mitochondria to produce energy to maintain the differentiation and functions (Chalmin et al., 2018). It has been reported that the activation of PPARγ coactivator-1α (PGC-1α) can enhance the antitumor efficacy of the infiltrating cytotoxic T lymphocytes (CTLs) by up-regulating fatty acid oxidation (Wan et al., 2020). In a mouse melanoma model, CD8^+^ T cells can enhance PPARα signal transduction and fatty acid catabolism for energy supply under the conditions of hypoxia and hypoglycemia in the TME (Zhang et al., 2017). Although this metabolic switch could increase the expression of coinhibitory molecule PD-1, it retained the effector function of CD8^+^ T cells. Moreover, PPARα agonists can further promote fatty acid catabolism and improve the ability of CD8^+^ T cells to arrest tumor progression.

**FIGURE 2:**
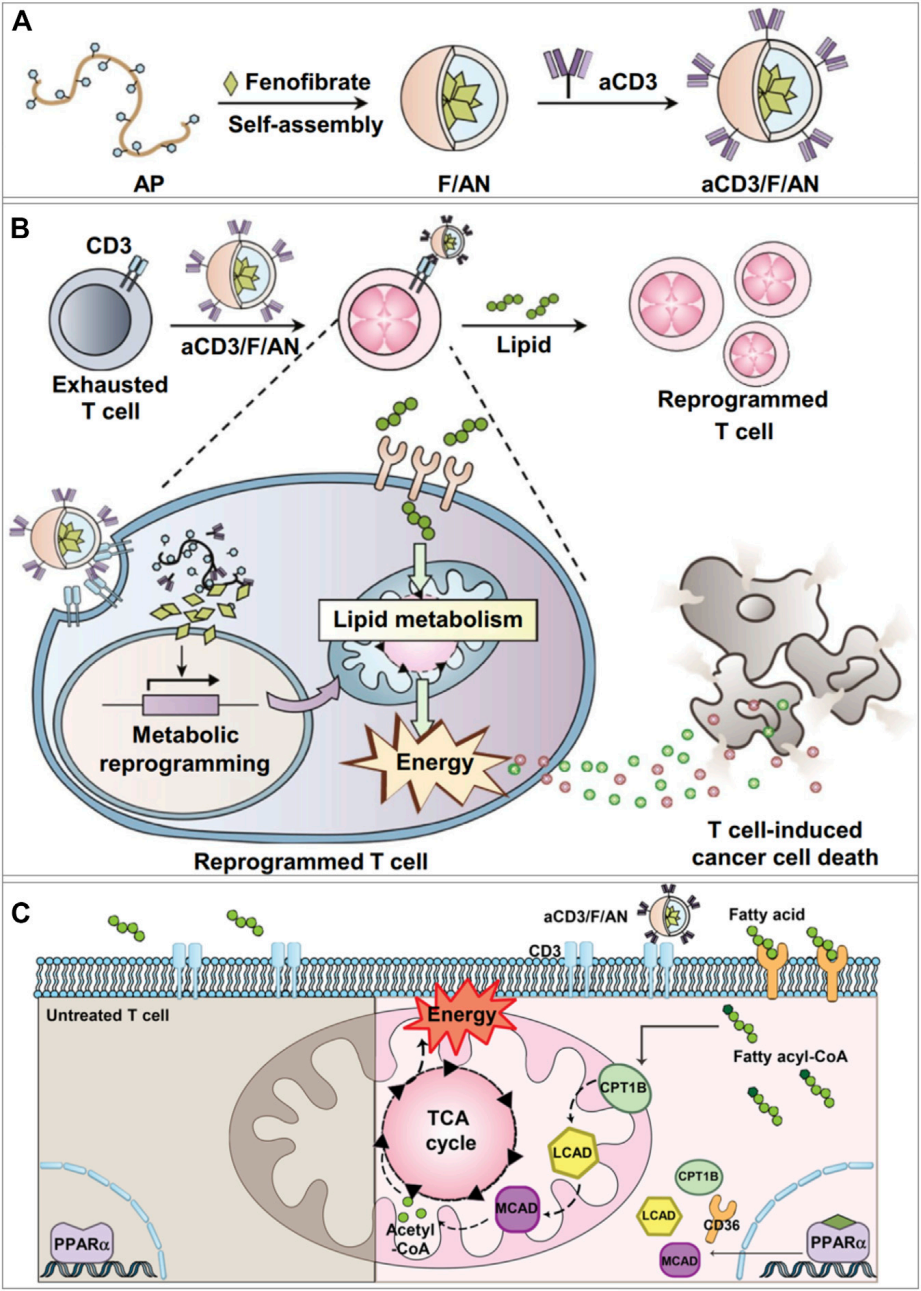
aCD3/F/AN mediated lipid metabolic reprogramming in T cells for immune-metabolic therapy (Kim D. et al., 2021). **(A)** Preparation of aCD3/F/AN nanoparticles. **(B)** The ability of T cells to kill tumors is enhanced by the activation of mitochondrial fatty acid metabolism. **(C)** Mechanisms of fatty acid metabolism activation in T cells. The left half of the schematic picture depicts the untreated T cells. In the right panel, it shows that aCD3/F/ANs enhanced the mitochondrial fatty acid metabolism by upregulating the expression of fatty acid metabolism-associated proteins, e.g., CD36, CPT1B, LCAD, and MCAD. Reprinted under the Creative Commons CC BY license.

To improve the functions of CD8^+^ T cells in the TME, Dongyoon et al. designed a targeted nanoparticulate system that can activate the anticancer activity of T cells through lipid metabolic regulation (Kim D. et al., 2021). The amphiphilic polyglutamic acid nanoparticles encapsulated fenofibrate (PPARα agonist) and the anti-CD3e f (ab′)2 fragment was modified on the nanoparticle surface to obtain the anti-CD3e-modified, fenofibrate-encapsulated nanoparticles (termed aCD3/F/ANs) (Kim D. et al., 2021). The aCD3/F/ANs efficiently targeted T cells by binding with CD3 receptor (Figure 2A), which is a marker of mature T cells preserved in various tumors. PPARα and the downstream genes related to fatty acid metabolism were significantly upregulated, and mitochondrial functions were enhanced in T cells by the treatment of aCD3/F/ANs (Figures 2B,C). Meanwhile, in the glucose-deficient environment mimicking the TME, aCD3/F/ANs promoted T cell proliferation by activating fatty acid metabolism. The tumor-bearing mice with aCD3/F/AN treatments showed the increased infiltration of T cells, enhanced production of anticancer cytokines, and prolonged survival time.

Chimeric antigen receptor (CAR)-T cell therapy has shown remarkable success in the treatment of B-cell malignant tumors, while the efficacy of CAR-T cell therapy in the treatment of solid tumors is limited (Rafiq et al., 2020). CAR-T cell therapy combined with proinflammatory cytokines or immune checkpoint inhibitors has gained progress in the preclinical and clinical treatment of some solid tumors, but there are still major challenges, e.g., the viability of CAR-T cells in the TME (Li et al., 2018). Regulating the lipid metabolism of CAR-T cells in the TME can enhance their antitumor ability. Cholesterol plays an important role in maintaining the function of T cells, and cholesterol in the cell membrane facilitates to cluster T cell receptors (TCR) and form immune synapses (Zech et al., 2009; Molnar et al., 2012). Increasing the content of cholesterol on the cell membrane may promote the antitumor effect of T cells. For instance, avasimibe, an inhibitor of the cholesterol-esterification enzyme acetyl coenzyme A acetyltransferase 1 (ACAT1), could increase the content of plasma membrane cholesterol, promote TCR clustering, and thus improve the antitumor activity of T cells (Yang et al., 2016). Therefore, cholesterol metabolism could serve as a potential target for improving the antitumor activity of CAR-T cells. To achieve efficient drug delivery, Hao et al. developed a liposomal system for delivering avasimibe, a metabolism-modulating drug, onto the T cell membrane surface by lipid insertion without interfering with T cell function (Hao et al., 2020). The liposome-encapsulated avasimibe inhibited the function of ACAT1 in CAR-T cells through both autocrine and paracrine effects, and thus increased the cholesterol level on the CAR-T cell membrane, promoted the rapid TCR clustering, and enhanced the continuous activation of T cells, which significantly improved the therapeutic effect of adoptive T cells in solid tumors.

CD8^+^ T cell-based immunity can be improved by enhancing the intratumoral T cell infiltration and activation. *In-situ* activation of the infiltrating T cells by nanomedicine is a promising immunotherapeutic method, and immune checkpoints related to lipid metabolism are the potential drug targets. However, CD8^+^ T cell-targeting delivery systems are still under-explored. A recent study revealed that the exosomes derived from CAR-T cells had strong anti-tumor effects (Fu et al., 2019). It suggested CAR-T cell-derived exosomes had the potential tumor homing ability. Exosomes as a biomimetic delivery carrier have recently attracted great attention, which is believed to be able to home to their cells of origin for a targeting purpose (Qiao et al., 2020). Therefore, exosomes could be a potential delivery strategy for T cell regulation.

### Macrophage-Targeted Nanotechnologies for the Enhancement of Tumor Immunity

**FIGURE 3:**
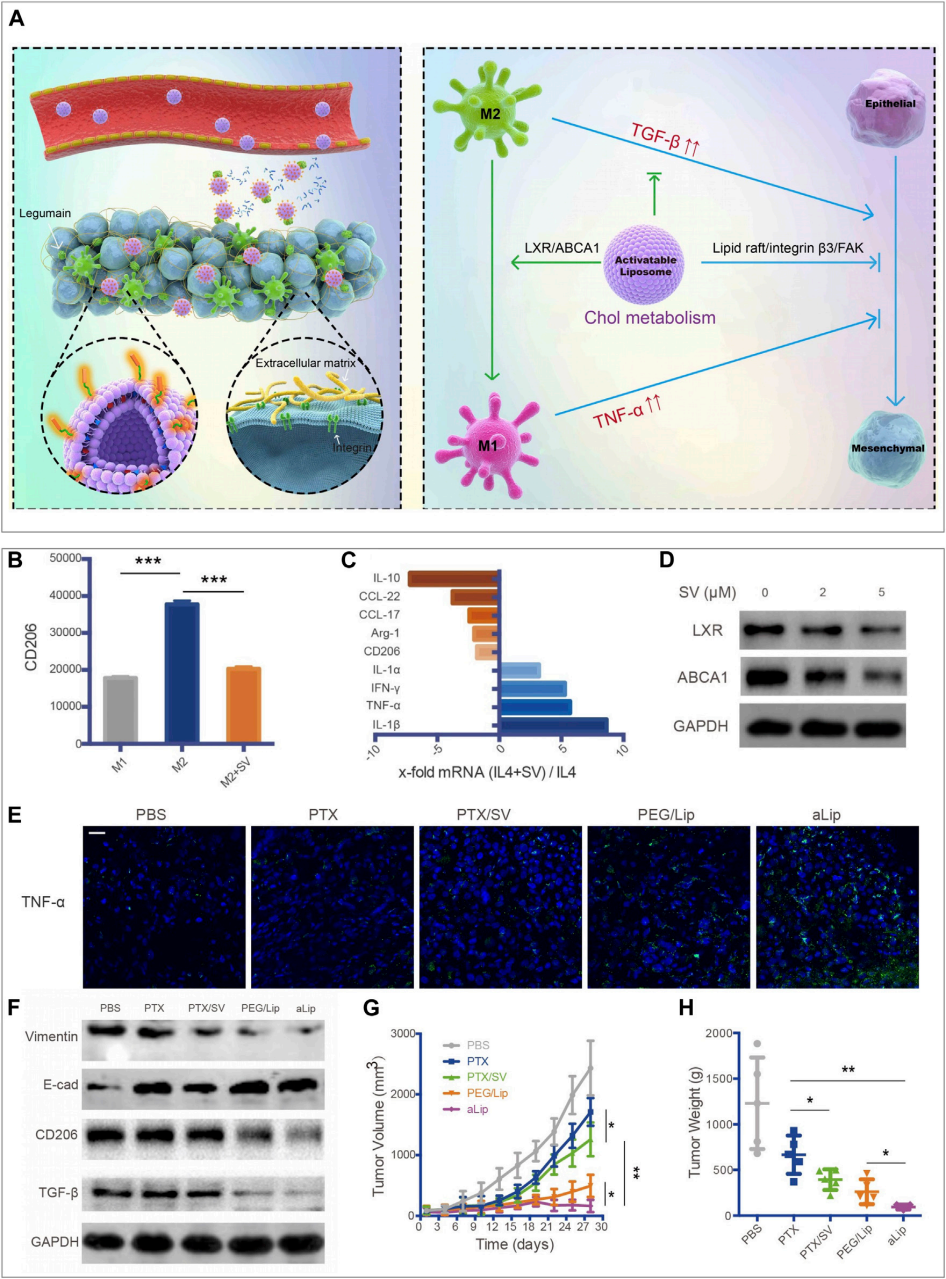
aLip mediated lipid metabolic reprogramming of macrophages for cancer therapy (Jin et al., 2019). **(A)** The preparation and function of aLip are illustrated. aLip could realize targeted drug delivery by responding to the overexpressed legumain in the TME and repolarize TAM through cholesterol metabolism. **(B)** SV reduced expressions of CD206 in M2 macrophages. **(C)** RT-PCR (Reverse Transcription-Polymerase Chain Reaction) detections of M1 and M2 related genes in M2 macrophages with the treatment of SV. **(D)** SV downregulated ABCA1 and LXR expressions in M2Φ. **(E)** aLip upregulated the expression of TNF-α in tumor tissues. **(F)** aLip suppressed the expression of CD206 and TGF-β in tumor tissues. **(G)** aLip inhibited the tumor growth in the subcutaneous A549T-xenograft model. **(H)** Tumor weight in the endpoint of treatments. Reprinted under the Creative Commons CC BY license.

TAMs promote tumor progression in varying aspects, including accelerating tumor angiogenesis, promoting tumor invasion and metastasis, and taming protective adaptive immunity (Noy and Pollard, 2014). TAMs may account for up to 50% of the tumor mass and be divided into a protumor phenotype M2 and an antitumor phenotype M1 (Solinas et al., 2009). Most TAMs are of protumor phenotype and contribute to the tumor immunosuppressive microenvironment. Once macrophages are recruited into the TME, the metabolic pathway in macrophages would be reprogrammed to adapt to the special environment. It has been reported that ovarian cancer cells could promote the outflow of macrophage membrane cholesterol, transform TAMs into the M2 phenotype, and promote tumor progression (Goossens et al., 2019). Therefore, regulating the cholesterol metabolism in macrophages could suppress the development of the tumor immunosuppressive microenvironment. Liver X receptor (LXR)/ATP binding cassette transporter A1 (ABCA1) plays a vital role in cholesterol homeostasis (Gabitova et al., 2014). Essentially, macrophages with the overexpression of LXRα showed the enhanced expression of the M2 phenotype genes and the decreased expression of the M1 phenotype genes (Liu et al., 2018). Furthermore, LXRα agonists could repolarize macrophages from M1 to M2 phenotype (Dou et al., 2019). Therefore, the LXR/ABCA1 axis in macrophages is a potential target for inhibiting tumor growth. Simvastatin, a 3-hydroxy-methylglutaryl coenzyme A (HMG-CoA) reductase inhibitor, has been wildly used for the treatment of dyslipidemia and prevention of stroke and heart attack. It has been reported that simvastatin could promote the repolarization of macrophages from M2 to M1 phenotype by suppressing the cholesterol-related LXR/ABCA1 axis, (Figures 3B–D) (Jin et al., 2019). Jin et al. developed a co-delivery multifunctional liposome loading with simvastatin (SV) and paclitaxel (PTX) for treating the drug-resistant lung tumor (Jin et al., 2019). The targeted liposomal system (termed aLip) was characterized by activatable cell-penetrating ability by responding to a tumor-associated enzyme legumain; aLip thus could enhance tumor delivery efficiency and achieve deep intratumor penetration **(** Figure 3A). The liposomes exhibited the therapeutic effect on re-sensitized the drug-resistant cancer cells and repolarization of TAMs, and thereby inhibiting lung tumor growth (Figures 3E–H **)**.

In theory, the pro-inflammatory M1 subtype exhibits more potent phagocytosis ability than M2 because the activated M1 are the major effector cells to phagocytose bacteria. In this sense, M1 are inclined to take up more nanoparticles. Therefore, it could be a challenge to efficiently achieve M2-like TAM subtype targeting delivery. On the other hand, the M2 population far outnumber M1 in the TME, which could offset the inferior uptake efficiency of M2.

## Perspectives

Immunotherapy represents a new era for cancer treatment and can be combined with chemotherapy, radiotherapy, and surgery. Off-target effects cause a lower-than-expected rate of the primary outcome of immunotherapy. The TME is a dynamic network; the cell population and phenotype are affected by the metabolic switch. There is a lipid-rich environment in many solid tumors, and lipid metabolism is conducive to tumor cell growth and immune response (Cruz et al., 2020). Because of the plasticity of immune cells, they can be directed toward an antitumor phenotype by regulating the metabolic checkpoints. The targeting lipid metabolism *via* nanotechnology provides a potential strategy to improve cancer therapy by regulating immune-metabolic signals. Although nanomedicines have been explored for tumor metabolism regulation (e.g., glycolysis and iron metabolism) (Wang et al., 2019; Lin et al., 2021), it is still an infant field of applying nanotechnology for lipid metabolism regulation for immunotherapy.

Lipid metabolism is highly diverse and complex due to the vast variety of endogenous lipid molecules and metabolites. The advance of understanding the regulatory pathways and the biological functions of the lipid molecules in different cells in TME will promote the further application of nanomedicine in regulation of lipid metabolism and immunity. For example, cholesterol may play a dual role in CD8^+^ T cells, and it either promotes anti-tumor responses or induces CD8^+^ T cell exhaustion (Yang et al., 2016; Ma et al., 2019). Therefore, how to fine-tune cholesterol hemostasis could be essential for precisely directing immune responses. For this purpose, nanotechnology-based drug delivery can provide a useful tool for elegant control of drug fate and action.

Nanotechnology can protect the encapsulated drugs from degradation and clearance, and thus prolong the drug half-life, as well as improve the solubility of drugs. Various immune cells in the TME play different roles in tumor immunity (Hao et al., 2021), and therefore, the targeting delivery to a specific group of immune cells is a prerequisite for precise regulation of the tumor immune microenvironment. *Via* surface modification with a targeting ligand that can specific binding to a certain receptor of the immune cells, the nanoparticles can selectively deliver drugs to the targeted cell type in the TME. It is a goal to achieve targeted delivery to a specific cell population (e.g., TAMs), and even a specific phenotype of a cell group (e.g., M2-like TAMs) in the TME. With the advance of nanotechnology-based delivery, precise regulation of lipid metabolism (e.g., targeting cholesterol metabolism in a specific cell type) will be feasibility, though there are still many unknowns to be explored, for example, to illustrate the relation between the drug biofate and the nanoparticle properties such as shape, charge, material, and surface functionalization.
